# Supplementary material for: Proteotranscriptomic Profiling of the Toxic Mucus of Kulikovia alborostrata (Pilidiophora, Nemertea)
Source: Toxins (Basel). 2024 Dec 26;17(1):5. doi: 10.3390/toxins17010005 (PMC11768458; doi:10.3390/toxins17010005)
Supplement: Supplementary file 1 [file toxins-17-00005-s001.zip › Supplementary File S4.zip!/ORF 006024 LINALB/230688690e3e92c6/aligs/c3lx4B_.98.alig.html]

Phyre 2 alignment of ORF\_006024\_LINALB with c3lx4B\_


|  |  |
| --- | --- |
| Return to main results | Retrieve Phyre Job Id |


|  |  |  |  |
| --- | --- | --- | --- |
| Job Description | ORF\_006024\_LINALB | | |
| Confidence | 19.27% | Date | Fri Apr 7 01:53:58 BST 2023 |
| Rank | 98 | Aligned Residues | 7 |
| % Identity | 57% | Template | c3lx4B\_ |


| PDB info | **PDB header:**oxidoreductase | **Chain:** B: **PDB Molecule:**fe-hydrogenase; | **PDBTitle:** stepwise [fefe]-hydrogenase h-cluster assembly revealed in the2 structure of hyda(deltaefg)  **PDB Entry:** PDBe RCSB PDBj |
| Resolution | 1.97 Å | | |

Show / Hide SS confidence

Show / Hide Conservation and Alignment quality

|  |  |  |  |  |  |  |  |  |  |
| --- | --- | --- | --- | --- | --- | --- | --- | --- | --- |
|  | Insertion relative to template ||  | Deletion relative to template |
|  | Catalytic residue from the CSA |
|  | |
| Detailed help on interpreting your alignment | |

  
  

|  |  |  |  |  |  |  |  |  |
| --- | --- | --- | --- | --- | --- | --- | --- | --- |
|  |  | 28 | . | 30 | . | . | . | . |
| Predicted Secondary structure |  | --- | --- | --- |  |  |  |  |
| Query SS confidence |  | --- | --- | --- | --- | --- | --- | --- |
| Query Sequence |  | C | C | P | G | Y | V | C |
| Query Conservation |  | --- | --- | --- | --- | --- | --- | --- |
| Alig confidence |  | --- | --- | --- | --- | --- | --- | --- |
| Template Conservation |  |  | --- | --- | --- | --- | --- |  |
| Template Sequence |  | C | C | P | G | W | I | A |
| Template Known Secondary structure |  | --- | --- |  |  |  |  |  |
| Template Predicted Secondary structure |  | --- | --- |  |  |  |  |  |
| Template SS confidence |  | --- | --- | --- | --- | --- | --- | --- |
|  |  | 129 | 130 | . | . | . | . | . |
|  |  |  |  |  |  |  |  |  |

|  |  |  |
| --- | --- | --- |
| Download: | Text version | FASTA version |

No model constructed - rank, confidence too low

  

  

---

Phyre is now FREE for commercial users!

All images and data generated by Phyre2 are free to use in any
publication with acknowledgement

Accessibility Statement

**Please cite:** The Phyre2 web portal for protein modeling, prediction and analysis

Kelley LA *et al.* *Nature Protocols*
10, 845-858 (2015)
[paper]
[Citation
link]

|  |  |
| --- | --- |
| © Structural Bioinformatics Group, Imperial College, London |  |
| Michael Sternberg |  |
| Disclaimer |  |
| Terms and Conditions |  |

Phyre2 is part of **Genome3D**
